# The effects of virtual reality and augmented reality technologies on students’ story retelling performance

**DOI:** 10.1371/journal.pone.0323445

**Published:** 2025-05-15

**Authors:** Bilal Şimşek, Betül Koparan

**Affiliations:** 1 Department of Turkish Language Education, Faculty of Education, Akdeniz University, Antalya, Türkiye; 2 Department of Turkish Language Education, Faculty of Education, Akdeniz University, Antalya, Türkiye; Father Muller Charitable Institutions, INDIA

## Abstract

This study aims to compare the retelling performance of two groups that engaged in reading activities with virtual reality and augmented reality texts. Furthermore, the results of the interventions using these technologies were compared with the results of the printed text reading activity. The study participants comprised 100 students aged 12–13 years studying in a secondary school. The researchers evaluated the students’ story-retelling performance through a rubric in the study. In the pre-test stage, the students performed a paper-based reading activity on the texts in the coursebook and their retelling performance was evaluated. In the post-test stage, the reading activities of the two groups were carried out with the intervention of virtual reality and augmented reality. While the pre-test results showed no significant difference between the groups, the post-test results indicated that the augmented reality intervention better supported the students’ retelling performance than virtual reality. However, there was no significant difference between the two groups in the sub-categories of setting and characters. Additionally, the virtual reality intervention did not create a significant difference in the sub-categories of characters, event/plot, problem, solution, and total score compared to the printed text reading activity. However, it produced better results in the setting sub-category than the printed text. A positive difference was observed in all sub-categories when the augmented reality intervention was compared to the printed text reading activity. AR showed greater benefits for retelling performance in this study, but further research is needed on long-term retention.

## Introduction

Reading encompasses the processes of understanding, interpreting, and analyzing written texts by individuals [1]. This process aims to improve students’ language skills, reading comprehension abilities, and thinking capacities [2,3]. The ultimate goal of reading, which is the ability to comprehend, is defined by the National Institute of Child Health and Human Development [4] as an essential skill for both students’ academic achievement and lifelong learning. While reading comprehension levels are typically measured through various test formats, evaluating individuals’ retelling performance can also be used as an alternative [5–12]. Retelling requires individuals to interpret and convey what they have read in their own words, reflecting the content of the text [13].

A review of the relevant literature reveals numerous studies examining the effects of retelling on students’ cognitive, affective, and social development [7,14–19]. These studies have found that retelling not only enhances students’ reading comprehension skills but also fosters the development of their creative thinking, critical thinking, and social skills [16,20–25]. Moreover, it has been determined that retelling contributes to students’ language development and supports their communication skills [26].

Technological advancements in recent years have introduced changes to reading materials, adding new dimensions to retelling. Alongside traditional printed texts, new text formats, such as digital texts [27–30], multimedia texts [31,32], and interactive texts [33,34]. Furthermore, augmented reality texts that combine printed and digital elements [35–38] and texts experienced solely in virtual reality environments [39–44] have also emerged. These contemporary text formats, enabled by technological developments, have the potential to transform the nature of traditional reading [45,46]. Consequently, understanding how these new text formats affect individuals’ reading processes has become an essential area of research. While several studies have compared reading experiences between printed and digital texts [47–50], research on the increasingly prevalent augmented reality (AR) and virtual reality (VR) texts remains limited. Most of these studies focused on comprehension [36,39] and vocabulary teaching [40,41]. Very limited studies exist on the impact of AR and VR technology on retelling the story. On the other hand, comparative studies on how AR and VR-based reading activities affect students’ reading processes have not been found in the relevant literature.

VR technology, one of the technological applications discussed in this study, provides users a three-dimensional, virtual, and interactive environment [51]. As Lin [52] explains, VR creates a three-dimensional virtual world through computer simulation and allows users to observe events and objects in three-dimensional space without limitation, providing sensory stimulation of sight, hearing, and touch. VR has been highlighted in numerous studies as a method that can enhance learning effectiveness in education [53–55]. However, it has also been suggested that while VR can be used as a tool in language classrooms, it faces challenges related to its technical configuration and pedagogical foundation [56]. For instance, VR can provide immersive experiences that engage students and enhance their learning, but it also requires a significant technical setup and may not be accessible to all students. Furthermore, Kwok et al. [57] have noted that improving reading comprehension skills in VR learning environments is challenging and remains understudied. Nevertheless, VR technology offers a different reading experience with the potential to create virtual environments suitable for reading content, as seen in the study of Acar and Cavas [39]. In this study, when students put on their VR glasses, they find themselves in a spacecraft and can go to different planets. Moreover, students can read short readings of these planets while navigating the 3D environment. Thus, students have a different reading experience than the traditional reading activity. In this context, it is important to determine how the reading experience affects students’ reading processes.

Another topic examined in this study is AR technology. AR is a technological tool that provides users with a new natural environment where they encounter both virtual and real components [58,59] and offers new options to enhance the innovativeness of instructional and learning applications [60]. Although the use of this technology in educational settings is relatively new, various positive outcomes have been reported. AR technology can potentially contribute to language teaching with its interactive format [61]. In the context of reading skills, its use is particularly encouraged in story reading and narration processes [62]. It has been stated that by providing students with learning opportunities based on understanding and experience, AR can help students internalize and retell stories more effectively [63]. However, context, interaction level, and technology integration must be considered when preparing AR-based texts to enable students to retell stories more meaningfully [64]. Not paying attention to these elements may negatively affect students’ story-retelling processes. Therefore, there is a need for current studies on the preparation of AR storybooks and their effects on students’ retelling performance. Understanding the potential effects of AR-based texts on students’ retelling performance is crucial for educators and developers in the field of education and technology integration.

While VR provides students with a vivid and three-dimensional environment for story-reading experiences [65], AR enriches the physical world with digital content [66]. Both technologies have the potential to create a more interactive environment in the learning process and to attract students’ interest and attention [67–79]. However, studies need to evaluate the reflections of this potential on students’ story-retelling performance. The current study will examine the effects of VR and AR-supported texts on the story-retelling process. Additionally, the results of interventions carried out with these technologies will be compared to the results of printed text reading activities. Thus, a broader knowledge base will be provided for educators, researchers, and technology application developers regarding these texts.

## Review of the literature

### Virtual reality technology

According to Fassi et al. [80], VR is a computer simulation of an actual situation in which individuals can interact with a virtual environment that does not exist through non-traditional interfaces such as goggles and helmets. VR is an advanced human-computer interface that simulates a realistic environment [81]. Participants can move within the virtual world, view it from different angles, reach out, grasp, and reshape it [82]. VR allows the creation of a virtual world using computer graphics and gives users genuine feelings [40,83–85]. In short, VR simulates a virtual environment that immerses users to the extent that they feel “a sense of being there” [86].

VR technology has enabled innovation across various fields [87]. First emerging in the field of computer science in the 1960s, VR is now being widely adopted in many areas [88–91]. Recently, its use has become more prevalent in education and language learning [92–94]. Previously, physical environments and materials were used entirely in language teaching, but digital technologies have recently started to be used. As an up-to-date technology that combines physical and digital elements, AR also offers users a different experience. On the other hand, VR offers users a virtual environment by completely detaching from the physical environment. In this respect, users’ cognitive load and experience may differ from traditional and AR applications that combine traditional and digital applications. In addition, while other applications have a connection with the real world, users who use the VR application switch from the real world to the virtual environment for a while through VR glasses. This feature of VR technology distinguishes it from other technology-supported language teaching processes. Since VR technology is an up-to-date technology used in language teaching and allows the creation of environments in different contexts, it has recently attracted the attention of researchers, and it has been observed that the number of studies on this subject is increasing day by day [65,95–101].

The studies in the literature indicate that VR has great potential to enable immersive, interactive, and intuitive language learning [56,90,99,102,103]. The research shows that the use of VR in language instruction reduces users’ anxiety levels [104,105], promotes cognitive gains [106–108], and provides a natural environment for practising speaking [44,109,110], reading [111,112], listening [113], writing [114], communication skills [115], critical thinking [116], and vocabulary knowledge [117–119]. However, challenges must be addressed in optimising VR-supported language education [101]. The challenges encountered in the use of VR in language teaching include internet connectivity issues and the lack of accessibility to the necessary devices for using VR [120–122], the inability to access VR platforms due to firewalls [123], occasional usage difficulties [121,124], the potential for cognitive and perceptual overload if VR-based learning activities are not adequately designed [125–128], the occurrence of symptoms such as nausea and dizziness in some students using VR [129], and the potential increase in workload [121,124,130].

The studies on VR-based reading environments have presented both positive and negative findings. It is argued that virtual reality offers an innovative approach to education, providing students with more effective experiences of texts [39,63,131,132]. VR strengthens the interaction with texts by presenting three-dimensional representations of the characters, events, and settings, helping individuals establish a deeper connection with the text [133]. Students can better understand the details within texts through the immersive learning experience in VR environments and can create more creative narratives by adding their own interpretations and perspectives during the retelling process [134,135]. With these aspects, VR-based reading environments are believed to contribute significantly to developing students’ retelling skills by providing enriched learning experiences in both cognitive and affective domains. On the other hand, Rau et al. [136] found no significant difference between VR/AR and traditional reading in their study that measured comprehension using a test. Additionally, Baceviciute et al. [137] compared students’ performance on a text read in a VR-supported environment and concluded that while the VR group performed better in information transfer, reading in VR was more cognitively demanding and less efficient in terms of time.

While the potential of VR in language learning is promising, there is still much to be explored. The literature suggests limited knowledge about VR’s effectiveness for learning [53,94]. Furthermore, the effects of VR on language teaching has not yet been sufficiently researched [57]. Compared to other language skills, studies on using VR for reading skills appear to be more limited [138]. Given VR’s unique opportunities for learning content, further research on VR-based reading environments is desirable and necessary. This need for further exploration should pique the curiosity and interest of educators, researchers, and practitioners in language education and technology.

### Augmented reality and augmented reality storybook

Augmented Reality (AR) is a technology where virtual information is overlaid onto real-world images, creating an interactive space for users to explore and facilitating learning [58] Unlike VR, AR is not designed to isolate the user from the real world [139]. While VR expects the user to experience a computer-generated virtual environment, AR expects the addition of information and images to be incorporated into a real-world environment by the system [140]. In this context, AR can be briefly explained as a technology where virtual objects are merged with the real-world environment [141,142]. The advantage of this technology is its ability to overlay virtual resources such as 3D images, animations, and videos onto the context and to support the static text encountered by the reader with a comprehensive virtual environment [143]. This allows for creating immersive experiences, providing the reader with interactive and enriched environments.

The beginnings of AR technology can be traced primarily to its use for educational purposes in aircraft engineering [144] and surgery [145]. For many years, the use of AR was limited to experimental tasks in academic laboratory settings, maintenance and repair activities [146–150]. However, today, AR is applicable in a wide range of areas. Education and language learning are among the current domains of AR application. It has been observed that AR is making various contributions to educational processes, and specific applications utilizing this technology are beginning to be introduced, particularly in language instruction [62].

When the relevant literature is examined, it is seen that AR-based environments in language teaching support learning performance [151–153], develop spatial thinking skills [154–157], increase motivation and interaction [158], improve vocabulary learning [139], make the process of learning and memorizing concepts more efficient [159], increase reading comprehension level and enrich learning environments [65]. On the other hand, some studies have revealed that while AR technology provides various benefits for language learning, it is not yet fully ready to be integrated into language classrooms [160] and that pedagogical strategies for its implementation need to be developed, and some points need to be considered in practice [161–163].

Regarding the potential of AR in education, it has paved the way for its integration into language teaching in different fields. One of the AR-supported teaching materials developed in this process is AR books. AR-based reading environments emerge as an innovative approach to education [164–166]. AR books are one of the instructional tools for the new technology that can be experienced using a computer, head-mounted display, smartphone, or tablet, and present information in different formats such as sound, digital content, 2D or 3D animation, and video [36]. Due to the educational and entertaining features of these books, they have become more widespread, especially for children, and research has shown that they provide students with the ability to understand better and interpret texts [66,167,168]. Furthermore, research on AR books in the relevant literature has shown that these books provide a more interactive space for storytelling by encouraging the use of touch in the exploration of story content [169]; they are exciting, engaging, and fun, and also facilitate the remembering of the story, expand the ability to empathize, and promote active reading [170–174].

The research on the effects of AR on reading skills has revealed that AR books provide an interactive environment that enriches individuals’ reading experiences by allowing the integration of virtual objects with the natural world [175,176]. Such environments deepen students’ interaction with the text and make the concepts of storytelling more concrete [177]. In this respect, this application has significant potential in terms of developing retelling skills [62,178], helping students better comprehend the details and meanings of the stories they tell [179], and enabling them to develop more creative and comprehensive discourse when retelling texts [106,169]. On the other hand, some studies have found that AR books do not make a difference in simple comprehension levels compared to printed books [168], and there is no difference between printed texts and AR books when students’ comprehension levels are evaluated [177]. Considering all the results, it is seen that AR-based reading environments offer significant opportunities for the development of reading comprehension and retelling skills. Therefore, this potential should be best realized through carefully designed applications, and current studies on this topic should be encouraged.

### Retelling

*Retelling* is the process in which readers, after reading a text, convey the main ideas, characters, and events in their own words to comprehend better the information [180]. Retelling enables students to recall the details within the text and make more connections [181]. Additionally, this process aims to facilitate students’ interaction with the text and reinforce the meaning they have derived from it [26]. Therefore, it can be said that retelling not only promotes the internalization of knowledge but also develops students’ thinking skills [182]. Retelling is a practical skill that strengthens reading comprehension abilities [183,184].

Developing retelling skills can be facilitated by employing various methods and technologies. In this context, it has been observed that emphasizing essential concepts within the text using interactive applications on intelligent boards or tablets during the instructional process can contribute to developing this skill [185]. Additionally, using video and animations to engage in storytelling can make retelling more captivating for students [186]. These technological opportunities allow students to explore different perspectives and develop and enrich their retelling techniques through diverse and more interactive experiences. In this regard, various technological tools can be utilized to help students better understand the text [187–190]. Some of these applications have emerged in recent years with the rapid advancements in educational technologies, such as VR and AR. These applications have emerged as contemporary tools that can be used to develop story-reading and retelling skills.

VR and AR applications have significant potential for facilitating comprehension. These technologies can help students better understand the events and characters described in the texts by making the content more concrete [7]. During the story reading process, students can observe the settings and events of the story in two or three dimensions through AR applications [191,192]. On the other hand, VR allows students to fully immerse themselves in the narratives, triggering their imagination and enabling them to become a part of the story [65]. These interactive experiences can give students more sensory experiences during retelling, making learning more enjoyable and effective. In this context, VR and AR technologies can significantly improve students’ retelling skills by contributing to better internalising the content.

The literature on the use of VR and AR to develop retelling skills is limited, but these technologies have significant potential [62,112,178,193,194]. These technologies have been found to facilitate more active student engagement in the learning process, enhance comprehension and memory skills, strengthen social interaction, and develop creative thinking abilities [195]. Many studies have reported that when students experience VR [196–202] and AR [203–207] environments, their connection to storytelling is strengthened, and these experiences promote deeper learning. However, some studies have also highlighted the potential adverse effects of VR [208–210], and AR [161–163] technologies. For VR, these issues include distraction from the narrative under certain conditions [211], increased cognitive load [212,213], and technical problems that can distract users [214]. Additionally, the psychological effects of VR on users need to be further investigated [208], and VR should be used in a balanced manner [215]. These factors may negatively effects the retelling performance of readers after using VR. For AR, issues such as complex interactions when users have to use multiple devices [216], increased anxiety levels in students [217], usability problems [191,218], the additional workload for teachers [219], and cognitive overload [191,220] have been reported. These factors may also negatively affect students’ retelling performance.

Based on the studies conducted, it can be said that VR and AR applications have the potential to significantly improve students’ retelling performance, offering a promising future for education. However, it is crucial to acknowledge the challenges and limitations that must be considered when using these technologies. On the other hand, it can be said that the studies on using VR and AR applications to develop retelling skills are limited. In this context, the current study aims to observe the effect of VR and AR-supported texts on retelling the story during the students’ retelling process and to provide recommendations based on the results. In the study, the rubric developed by Cruz de Quiros et al. [221] will be used to evaluate the students’ story-retelling performance. Thus, the effect of the texts prepared using VR and AR on both the total scores of the retelling performance and the sub-dimensions of the rubric, which are “setting when and where, characters, event/plot, problem and solution”, will be examined. This will provide us with more in-depth information about the effect of the texts supported by these technologies on students’ retelling performance.

### *The* current study

This study aims to compare the retelling performance of two groups that conducted reading activities with virtual reality and augmented reality texts. The study addresses the following research questions:

RQ1: What is the difference between the pre-test and post-test scores of the group participating in the printed text reading and virtual reality reading activities?
RQ2: What is the difference between the pre-test and post-test scores of the group participating in the printed text reading and augmented reality reading activities?
RQ3: What is the difference between the retelling performance of the group that participated in the virtual reality reading activity and the group that participated in the augmented reality reading activity?

## Methodology

### Participants

The participants were students attending a secondary school, ages 12–13. The students were willing to participate in the study. After obtaining the necessary permissions, the research process was initiated. One hundred students who voluntarily participated in the research process and studied in four different classes were randomly divided into Group 1 (26 girls and 24 boys) and Group 2 (27 girls and 23 boys). The students in the classes were randomly assigned to groups, and after the assignment, it was seen that balanced groups were formed in terms of gender. Group 1 participated in a reading activity using virtual reality, while Group 2 participated in a reading activity using augmented reality.

The principle of applicability is the main reason for including 100 participants within the research framework. There were no VR glasses in the practice school, but the researchers provided tablets used for AR-assisted readings. For this reason, considering the research’s applicability, 100 participants were included in the study. In addition, it was determined that most of the research conducted with VR, in particular, was conducted with a small working group in the context of applicability [112,136]. Among the 100 participants, not a single one was without access to technological devices (computers, tablets, smartphones, virtual reality devices, etc.). A staggering 64% of the participants owned a tablet, 78% owned a computer, and a whopping 86% owned a smartphone. These digital natives were adept at using their devices for a variety of purposes, with 84% using them for gaming, 74% for studying, 59% for entertainment, and 57% for communication. The data unequivocally demonstrated the students’ remarkable adaptability to technology. On the other hand, it was determined that the participants did not have a VR headset. In addition, the participants had no previous experience reading AR storybooks. However, the use of AR technology through tablets and the fact that students have used this technological device before may have provided them with convenience in reading activities with this device.

### Virtual reality setting and the augmented reality storybook

In this study, with teacher input, we chose the text “Little Dolphin” from the textbooks to measure the students’ retelling performance. This text covers the sub-dimensions of the rubric we used to evaluate the students’ retelling performance. Therefore, the researchers and the teachers considered it suitable for this study. Additionally, using a text selected from the textbook shows that these texts can be supported with virtual reality and augmented reality applications. For the VR-reading condition of the selected text, we developed a spatially adapted VR application using Unity3D. To provide environmental support, we also utilized the visuals from the “Little Dolphin” text in the students’ textbooks. This allowed us to place the reading text in an appropriate virtual environment as the context. The environment did not contain a character representing the students. We created a still and silent environment with the sea, a small dolphin, and a polluted beach. We only transferred the characters and locations from the text to the digital environment to ensure that the readers could only obtain information related to the text from the text itself. We positioned the reading text as an open book, spanning two pages, within the environment. During the reading, the students had no opportunity to interact with any elements in the environment, as they intended to focus solely on the text. The students read the text using the Oculus Quest 2, an independent VR headset.

To create the augmented reality storybook, we followed various steps. First, we determined which parts of the text would be supported by augmented reality. We then searched for video content to be used in these sections. We ensured that the augmented reality content integrated into the reading text would contribute to understanding the text by being consistent with the text. In this context, we prepared videos aligned with the flow of events in the text. We then recorded the audio for the video content that would replace the written sections of the text in a studio environment and integrated these audio recordings into the videos. When preparing the video content, we also considered the format that would support the students’ cognitive load and reading comprehension levels based on the study’s results by Şimşek et al. [222]. The duration of the digital content we prepared varies between 25–27 seconds. Therefore, we prepared the augmented reality content in a way that would not disrupt the integrity of the printed text and would contribute to the students’ understanding. After completing this process, we integrated the augmented reality content into the books using the ROAR application. This application ensures that the augmented reality content uploaded to the system matches the visuals in the books. Thus, the corresponding digital content is triggered by opening the ROAR application on a device such as a tablet or smartphone and bringing it over the visual in the printed book. The digital content triggered through this application only animates the visual in the text, while the written section continues to be visible on the application’s screen. In the current study, the students used the application on tablets.

### Procedures

This study was evaluated by the Akdeniz University Scientific Research and Publication Ethics Committee. The approval decision of the board numbered 879168 was sent to us in writing on 26.03.2024. In addition, application permission was obtained from the Directorate of National Education to carry out the research process in schools. In addition, a written informed consent form was requested from parents and students for participation. We obtained the necessary permissions and met with the school administrators where the implementation would take place. We then convened with the teachers, ensuring they were fully informed and involved in our study. We took the time to demonstrate to them how both virtual reality and augmented reality applications operate, fostering a sense of shared understanding and participation.

Respect for the students’ comfort was a key consideration in our study. We requested that the teachers be present in the classroom alongside the researcher during the research process, as we desired the students to feel more at ease. In the initial phase of the research, we aimed to test whether any difference existed in the retelling performance between the two groups. In this context, the students read the text “Little Dolphin” in print form and subsequently retold the text. We evaluated the students’ performances as a pre-test. We planned a five-week gap until the post-test phase, during which the teachers continued their regular instructional activities. After collecting the pre-test data, we conducted activities with one group using a virtual reality application and the other group using an augmented reality application, lasting two class periods (80 minutes for each group), with the goal of familiarizing the students with these applications. We did not utilize the text that was read in the pre-test during these activities. With the texts we determined, we enabled students to learn how to use these technological tools in VR and AR-supported reading processes. In VR, the researchers opened the reading environment, VR glasses were given to the students, and a reading experience was provided. The AR storybook reading processes explained how to use the tablets with the AR application open, and the students were asked to apply it. Similarly, in the final test phase, we opened the applications (application) and gave them to the students. This method was adopted so that the elements related to the applications did not create additional pressure on the students. Therefore, students were only expected to interact with the text. In the reading activities with different texts before the post-test, we verbally asked the students questions about the text and made practical speeches. We carried out these practices with groups of 25 people, and each group had two lesson hours (80 minutes). Additionally, we did not intervene with the groups in any other way outside of these activities. After the break, one group participated in a reading activity with the virtual reality application, while the other group participated in a reading activity with the augmented reality application. We asked the students, who read the same text through different technological applications, to retell the text. We then recorded the students’ retelling performances and proceeded to the analysis phase.

### Data analysis

In the study, the researchers evaluated the students’ story-retelling performances using the rubric proposed by Cruz de Quiros et al. [221]. This rubric consists of the following indicators: Setting, when and where, characters, event/plot, problem, and solution. The possible score range for a story is 0–15. After collecting the data, we first conducted normality tests to determine the appropriate analyses to be performed. After the analysis, we examined the Skewness and Kurtosis values and determined that the data showed a normal distribution. To analyze the pre-test scores of the two groups participating in the paper-based reading activity, we used independent group t-tests. Following the post-test, we conducted paired sample t-tests for the within-group evaluations. To compare the groups, we used independent group t-tests to find the difference between the post-test scores of the group that participated in the virtual reality reading activity and the group that participated in the augmented reality reading activity. Finally, in cases where the difference was significant, we calculated the effect size. We chose to use Cohen’s d to determine the effect size, which underscored the significance of our findings.

## Results

We conducted the pre-test with printed texts at the end of the reading activities. The post-test was administered after the two groups’ reading activities with virtual and augmented reality interventions. The pre-test results showed no statistically significant difference between the groups at a 95% confidence interval. However, the post-test results provided data indicating that the augmented reality intervention supported the students’ retelling performance more than the virtual reality intervention. Table 1 presents the means, standard deviations, and analysis results for the groups’ pre-test and post-test scores.

**Table 1:** Pre- and post-test means and standard deviations for retelling.

			Pre-test		Post-test		
	Group	N	Mean	Sd	Mean	Sd	*t*
Total score	VR	50	9.44	2.562	9.87	2.13	-1.915
	AR	50	9.8	2.579	11.36	2.501	-6.094*
Setting	VR	50	1.86	.585	2.16	.584	-3.130*
	AR	50	1.88	.606	2.1	.646	-2.292*
Characters	VR	50	1.94	.585	2.02	.588	-1.159
	AR	50	2	.606	2.18	.595	-3.130*
Even/plot	VR	50	1.92	.600	1.88	.520	.531
	AR	50	1.98	.552	2.28	.536	-4.583*
Problem	VR	50	1.92	.744	1.98	.706	-.704
	AR	50	2.07	.735	2.43	.735	-2.717*
Solution	VR	50	1.8	.801	1.83	.697	-.375
	AR	50	1.89	.899	2.37	.747	-3.855*

*p < .05

Based on the total scores, it was determined that the virtual reality intervention, while not creating a significant difference between the pre-test and post-test (t = -1.915, p < .05), showed potential for enhancing students’ retelling scores. However, the augmented reality intervention was found to significantly improve the students’ retelling scores (t = -6.094, p < .05). According to the within-group evaluations, after the augmented reality intervention, the students’ retelling performance significantly improved in all categories, including the total score, compared to the pre-test. On the other hand, after the virtual reality intervention, the students’ performance only improved in the “setting” sub-category (t = -3.130, p < .05). There were no significant differences in the “characters” (t = -1.159, p > .05), “event/plot” (t = .531, p > .05), “problem” (t = -.704, p > 05), and “solution” (t = -.375, p > .05) sub-categories compared to the pre-test. The Independent Groups t-test results for the pre-test and post-test are presented in Table 2 and Table 3, respectively.

**Table 2:** Results for the comparison of pre-test retelling scores of VR and AR groups.

	t	*p*
Total score	-.700	.486
Setting	-.150	.881
Characters	-.503	.616
Even/plot	-.520	.604
Problem	-1.013	.313
Solution	-.528	.599

**Table 3:** Results for the comparison of post-test retelling scores of VR and AR groups.

	t	*p*	Cohen *d*
Total score	-3.207	.002	.641
Setting	.487	.627	–
Characters	-1.351	.180	–
Even/plot	-3.785	.000	.757
Problem	-3.119	.002	.624
Solution	-3.735	.000	.747

During the pre-test phase of the study, we had the two groups read printed texts to evaluate their story-retelling performance. After reading the text, the students retold the story, and we conducted the pre-test evaluation. The results show that in the absence of any intervention, there was no significant difference between the two groups’ retelling performance (t = -.700, p > .05). This finding applies to the total score and all sub-categories. It’s important to note that before the virtual reality and augmented reality interventions were implemented, the two groups had remarkably similar story-retelling abilities, as evidenced by the non-significant difference in their pre-test scores, reinforcing the robustness of our findings.

The post-test results of the study show that the students who participated in the augmented reality-supported reading activity had significantly higher story-retelling performance compared to the students who participated in the virtual reality-supported reading activity (t = -3.207, p < .05). This is also the case for the “event/plot” (t = -3.785, p < .05), “problem” (t = -3.119, p < .05), and “solution” (t = -3.735, p < .05) sub-categories. There is a statistically significant difference in favour of the augmented reality intervention in these sub-categories compared to the virtual reality intervention. However, for the “setting” (t = -2.144, p < .05) and “characters” sub-categories, there is no significant difference between the two groups. Additionally, we calculated the effect size for the categories where we found a significant difference. The Cohen’s d effect size is.641 for the total score,.757 for the event/plot,.624 for the problem, and.747 for a solution. These results indicate that the augmented reality intervention supported the students’ story-retelling performance more effectively, particularly in event/plot, problem, and solution, than the virtual reality intervention. While the “p” value tells us that there is a statistically significant difference between the groups, the effect size shows us how important this difference is from a practical point of view. When the effect sizes calculated in the present study are evaluated, it is seen that there is a moderate effect size in all of the titles. When these results and the results of the in-group assessment are evaluated together, it can be said that AR is a more appropriate tool to increase students’ retelling performance than VR. These results apply to the current research design.

Based on the information provided above, the research questions can be answered as follows:

RQ1: The results of the reading activity conducted through virtual reality, while not showing a significant difference in the students’ retelling performance compared to those conducted with printed texts, do suggest a potential for positive effect. The virtual reality intervention positively affected the students’ performance in the setting sub-category, hinting at its future potential in education.
RQ2: The reading activity conducted through augmented reality, as evidenced by our research, better supports the students’ retelling performance than the reading activity conducted with printed texts. This finding, validated across the setting, characters, event/plot, problem, and solution sub-categories, instils confidence in the robustness of our research.
RQ3: The reading activity conducted through augmented reality better supports the students’ retelling performance compared to the reading activity conducted through virtual reality. This applies to the event/plot, problem, and solution sub-categories. There is no difference between the groups in the setting and characters sub-categories.

## Discussion

Our study takes a unique approach, focusing on the effects of VR and AR applications on students’ story-retelling performance. We designed texts suitable for both environments, allowing students to read from their textbooks using VR and AR applications. In the pre-test, students read the texts with printed materials, and in the final test, the two groups read the texts with the two technological tools. We evaluated the students’ retelling performance in depth, considering their total scores and their scores on the sub-categories of setting, characters, event/plot, problem, and solution rather than as a single dimension.

Results related to the first question of the research showed that virtual reality intervention did not significantly affect students’ story-retelling performance compared to printed texts. Additionally, the virtual reality intervention did not create a significant difference in the categories of characters, events/plots, problems, and solutions. While there are a limited number of studies on this subject in the literature, the results do not fully support each other. The results generally indicate that the VR intervention did not produce successful results in the context of reading comprehension. In this context, Flores-Gallegos et al. [223] concluded in their study that VR environments did not significantly affect reading performance. Similarly, in the study by Çoban et al.[209], printed texts were found to be more beneficial than virtual reality in terms of both reading comprehension and retelling performance. In the study, information-based questions about the concrete responses in the story were generally used to measure the participants’ retelling performance. However, in the study by Çoban et al. [209], the VR content was modelled and used only as a book and an appropriate virtual environment for the context of the text was not created. This may explain why the VR intervention in our study supported the students’ retelling performance related to the setting. Indeed, we found that the students who participated in our study provided more detailed information about the setting after the VR intervention. In the study by Baceviciute et al. [137], a virtual environment appropriate to the context was created, and it was revealed that virtual reality could successfully support information transfer. In this framework, the study by Kaplan-Rakowski and Gruber [112] also showed that VR could support reading comprehension. In the study, two groups reading subtitles with VR and video experiences were compared, and the results showed that the VR group had statistically significantly higher reading comprehension scores than the video group. These results may differ from those of other studies due to using videos instead of printed texts. However, it was determined that the VR intervention increased motivation, and there was no difference between the groups regarding cognitive load [112]. Therefore, these factors may also have supported the comprehension process. We think it is still too early to answer clearly how VR intervention affects reading comprehension or retelling performance. Indeed, the studies mentioned in this section were generally conducted with university students. In this respect, conducting the current study with middle school students is also very important for understanding the effects of virtual reality on different groups.

Another topic addressed in the study is the effect of augmented reality intervention on retelling performance. The results prove that the reading activity performed with augmented reality better supports students’ retelling performance than reading activities with printed texts. This result is also valid for setting, characters, event/plot, problem, and solution categories. Many studies in the literature have shown that augmented reality applications support learning performance [152,224], positively affect academic achievement [225,226], are engaging [227,228], increase students’ motivation for learning [157,229], and provide users with a pleasant learning environment [230]. Therefore, the characteristics of augmented reality-supported texts may have contributed to understanding the text and the retelling processes. Indeed, there is ample evidence that augmented reality storybooks increase users’ comprehension performance [44,66,167,168]. Additionally, studies have been conducted on students’ story-retelling performance. These studies determined that the augmented reality intervention increased students’ story-retelling performance [36,62,178]. However, considering the sub-dimensions of retelling, it can be said that our study results are different. Indeed, Danaei et al. [36] revealed that the augmented reality intervention may not make a difference in describing the story’s theme, characters, and setting. On the other hand, Liu et al. [178], similar to the results of the current study, determined that students could perform better in describing the story’s setting after the augmented reality intervention. This difference may be due to the diversity of the augmented reality content. Indeed, more subjective opinions may be at the forefront when preparing augmented reality storybooks with unclear design principles. This leads to the use of multimedia content without a standard. Therefore, differences may arise in the results of studies with a similar research framework. However, when the mentioned studies are examined, it has been determined that after the augmented reality intervention, students performed better in describing the overall story and the sequence of events [36,62]. These results support the current study. In conclusion, it can be said that the augmented reality intervention supports students’ retelling performance.

The current study’s findings indicate that the read-aloud activity implemented with AR was more supportive of students’ retelling performance than the read-aloud activity implemented with VR. This was the case for the event/plot, problem, and solution categories. However, there were no differences between the groups in the setting and characters sub-categories. This may be attributed to the fact that the character and the setting were represented in the virtual reality environment. Therefore, it can be argued that creating a context-based background supports students’ retelling performance while reading the text. The results show that the AR intervention is more effective and that this effect is a moderate effect size. Considering the in-group evaluations, using AR storybooks in classroom activities can encourage an increase in students’ performance in retelling the story based on the determined impact sizes. It is known that AR integration into textbooks currently used in schools is easier than VR. In addition, AR applications can be used with more widely used technological tools such as smartphones and tablets. For this reason, the use of AR applications in classroom reading activities can be adopted based on the effect sizes determined regarding the retelling performances of the students and the ease of use.

When the results are evaluated overall, the augmented reality intervention stands out. One of the possible reasons for this may be that adding multimedia content such as sound, video, and interaction to the text enriches the learning experience [231,232]. The presence of stimuli such as sound and video in the augmented reality storybook, which are absent in the virtual reality environment, maybe a reason for this difference. Indeed, receiving information through different channels, such as auditory and visual, compared to processing it through a single channel, contributes to more efficient learning and retention [27]. Thus, the multimedia content may have reduced the cognitive load and increased the comprehensibility of the story [29]. Moreover, there is evidence that virtual reality can increase the cognitive load in the context of reading [137], while augmented reality can reduce it [222], which may be another reason for the difference between the two groups. Additionally, most studies on augmented reality in the context of reading skills have reported positive results regarding story retelling [36,62,178]. In contrast, virtual reality studies have found some negative results [209,223]. The less frequent use of virtual reality, students’ unfamiliarity with reading in a virtual environment, and the requirement to use a head-mounted display may present an unusual reading experience for students [209]. On the other hand, the use of printed texts in augmented reality reading experiences may make students feel more comfortable.

## Conclusion

This study explored the effect of reading activities conducted with VR and AR applications on students’ retelling performance. To this end, we formed two groups and first conducted a reading activity with printed texts to evaluate the students’ retelling performance. Subsequently, the groups engaged in reading activities with VR and AR interventions, and their post-test scores were determined. The results showed that the VR intervention did not significantly differ from the printed texts. However, the AR intervention proved to be a practical and effective tool, particularly in the setting sub-category, demonstrating its usability in the context of traditional texts. The reading activity conducted with augmented reality significantly better supported the students’ retelling performance compared to the reading activity with printed texts. This finding was valid for the setting, characters, event/plot, problem, and solution sub-categories. When we looked at the difference between the groups, we determined that the reading activity with augmented reality better supported the students’ retelling performance than the reading activity with virtual reality. This was also true for the event/plot, problem, and solution sub-categories. There was no difference between the groups in the setting and characters sub-categories. The current study is essential in revealing the contributions of current technological applications to students’ story-retelling performance. Thus, this study may encourage reading activities using augmented reality, as it demonstrates the practicality and usability of these applications in the context of traditional texts. Augmented reality applications can be used through many tools, such as smartphones, tablets, and smart boards. With the FATIH project, which was carried out in the context of equal opportunity in education in Turkey, tablets were distributed to students and smart boards were provided to schools. In addition, various infrastructure and equipment needs of schools were met. In this respect, AR applications can be used more widely in Turkey’s classroom environment than VR applications. On the other hand, schools still lack infrastructure and technological tools. These classes are unsuitable for AR and VR applications in reading education. In these classes, applications can only be carried out within the means of the teachers and students. Additionally, the limited number of studies in the literature on the technological applications tested in the research, especially virtual reality, makes our results noteworthy.

## Limitations and recommendations

This study has some limitations. First, the age range of the participating students is 12–13 years old. Therefore, the results can be considered limited to this age range. Future studies can focus on this age group and different age groups, allowing for more general comments on the topic. In addition, using eye-tracking technology in future research will provide more detailed data on reading processes. This study used only quantitative data, but it is recommended that qualitative data be collected to get in-depth information about students’ experiences in new studies. Additionally, the sample size is limited to 100 students. Although large study groups are not often encountered in research on current technological tools, more studies are needed to generalize the results. Individual differences in students’ cognitive load in this study may also have affected their comprehension. These differences may also be taken into account in future studies. In addition, it may be necessary to draw attention to the issue of motion sickness in VR, especially in studies. Obtaining data on this situation from students in the studies to be carried out will provide more insight when evaluating the students’ performance. The current study is limited to a text from the 7th-grade native language textbook currently in use. Due to the need to prepare both virtual reality and augmented reality content, more texts were not prepared. In addition, this text is a narrative text. Therefore, future studies can adopt a similar research framework using different or multiple texts, contributing to the generalizability of the relevant results. Moreover, the preparation of the text used in the current study based on the textbook has shown that current technologies can be used in school settings. In this context, it is recommended that future research focus on integrating these technologies into school-used course materials. Additionally, studies should be conducted to establish design principles that answer questions such as how to prepare a reading text in a virtual environment or augmented reality texts. In addition, the current comparison is limited to the digital content used in this study. It should not be ignored that the results may be affected when different virtual reality and augmented reality contents are created. Finally, the focus of this study is limited to evaluating students’ retelling performance. It is recommended that future studies include reading comprehension tests to provide more in-depth insights into students’ reading comprehension.
